# Blockade of NF-κB and MAPK pathways by ulinastatin attenuates wear particle-stimulated osteoclast differentiation *in vitro* and *in vivo*

**DOI:** 10.1042/BSR20160234

**Published:** 2016-10-27

**Authors:** Jiang-Ying Ru, Hai-Dong Xu, Dai Shi, Jun-Bo Pan, Xiao-Jin Pan, Yan-Fen Wang

**Affiliations:** * Department of Orthopedics, The First People's Hospital of Yangzhou City, Second Clinical School of Yangzhou University, Yangzhou 225000, China; † Department of Orthopedics, Jinling Hospital, School of Medicine, Nanjing University, Nanjing 210002, China; ‡ Department of Pathology, The First People's Hospital of Yangzhou City, Second Clinical School of Yangzhou University, Yangzhou 225000, China

**Keywords:** aseptic loosening (AL), lipopolysaccharide (LPS), mitogen activated protein kinase (MAPK), nuclear factor kappa B (NF-κB), osteoclast, osteolysis, polymethyl-methacrylate (PMMA), ulinastatin

## Abstract

Ulinastatin, a urinary trypsin inhibitor (UTI), is widely used to clinically treat lipopolysaccharide (LPS)-related inflammatory disorders recently. Adherent pathogen-associated molecular patterns (PAMPs), of which LPS is the best-studied and classical endotoxin produced by Gram-negative bacteria, act to increase the biological activity of osteopedic wear particles such as polymethyl-methacrylate (PMMA) and titanium particles in cell culture and animal models of implant loosening. The present study was designed to explore the inhibitory effect of UTI on osteoclastogenesis and inflammatory osteolysis in LPS/PMMA-mediated Raw264.7 cells and murine osteolysis models, and investigate the potential mechanism. The *in vitro* study was divided into the control group, LPS-induced group, PMMA-stimulated group and UTI-pretreated group. UTI (500 or 5000 units/ml) pretreatment was followed by PMMA (0.5 mg/ml) with adherent LPS. The levels of inflammatory mediators including tumour necrosis factor-α (TNF-α), matrixmetallo-proteinases-9 (MMP-9) and interleukin-6 (IL-6), receptor activation of nuclear factor NF-κB (RANK), and cathepsin K were examined and the amounts of phosphorylated I-κB, MEK, JNK and p38 were measured. *In vivo* study, murine osteolysis models were divided into the control group, PMMA-induced group and UTI-treated group. UTI (500 or 5000 units/kg per day) was injected intraperitoneally followed by PMMA suspension with adherent LPS (2×10^8^ particles/25 μl) in the UTI-treated group. The thickness of interfacial membrane and the number of infiltrated inflammatory cells around the implants were assessed, and bone mineral density (BMD), trabecular number (Tb.N.), trabecular thickness (Tb.Th.), trabecular separation (Tb.Sp.), relative bone volume over total volume (BV/TV) of distal femur around the implants were calculated. Our results showed that UTI pretreatment suppressed the secretion of proinflammatory cytokines including MMP-9, IL-6, TNF-α, RANK and cathepsin K through down-regulating the activity of nuclear factor kappa B (NF-κB) and MAPKs partly in LPS/PMMA-mediated Raw264.7 cells. Finally, UTI treatment decreased the inflammatory osteolysis reaction in PMMA-induced murine osteolysis models. In conclusion, these results confirm the anti-inflammatory potential of UTI in the prevention of particle disease.

## INTRODUCTION

Total joint replacement (TJR) is a commonly orthopaedic procedure performed for the relief of pain from osteoarthritis, rheumatoid arthritis, posttraumatic arthritis and other end-stage joint disease [1,2]. However, wear particles, originating from the implant component, stimulate the release and phagocytic activity of monocytes and macrophages around periprosthetic tissue, and generate a host of proinflammatory cytokines, including tumour necrosis factor-α (TNF-α), MMPs, interleukin-1α (IL-1α) and IL-6 [3,4]. The resulting inflammatory reaction is widely considered to be the main triggering cause of periprosthetic osteolysis (PPO) and consequent aseptic loosening (AL), so-called ‘particle disease’, which may result in failure of the implant necessitating the revision of arthroplasty [5–8]. It has been found that pathogen-associated molecular patterns (PAMPs), including lipopolysaccharide (LPS) and lipoteichoic acid (LTA) mainly, could increase the inflammatory and osteolytic effect of wear particles *in vitro* and *in vivo* [9,10]. Moreover, PAMPs probably exist in periprosthetic tissue of some patients with AL, which is often found on implants retrieved from patients despite the lack of any clinical signs of infection [11–15]. In addition, the systemic circulation and the implant itself are also possible sources of bacterial PAMPs during AL.

Nowadays, medicines investigated mainly include bisphosphonates, macrolides and tetracyclines, which all have a certain inhibitory action on nuclear factor kappa B (NF-κB) signalling pathway and formation of mature osteoclasts (OCs). However, they all exist some defects hard to be neglected [16,17]. Urinary trypsin inhibitor (UTI), as a natural and classical type Kuniz broad-spectrum proteinase inhibitor secreted by human liver, can inhibit the produce of proinflammatory factors and down-regulate the activation of NF-κB signalling pathways. It is not only a standard treatment worldwide for the LPS-related diseases but also has anticancer and immunosuppressive actions with less side effects [18,19]. Regardless of many studies about UTI, the pharmacological effect of itself has not been fully understood, and little is known about its anti-inflammatory effect in the LPS-related periprosthetic osteolysis. The present study examined the effect of UTI on the expression of inflammatory mediators in LPS/polymethyl-methacrylate (PMMA)-stimulated Raw264.7 cells and also investigated the action of UTI on the PMMA-induced murine femoral osteolysis model. With the data, the present study was extended to explore the role of UTI on the NF-κB and mitogen activated protein kinases (MAPKs) signalling pathway as a plausible molecular mechanism.

## MATERIALS AND METHODS

### Reagents and PMMA particles preparation

UTI was provided by Mochida Pharmaceutical Company. Antibody directed phosphorylated inhibitory-κBα (p-I-κB), p-ERK (1/2), p-MEK, p-JNK antibodies were obtained from Cell Signaling Biotechnology. Anti-I-κB, anti-NF-κB (p65 subunit), anti-ERK (1/2), anti-MEK, anti-JNK antibodies and anti-actin antibodies were purchased from Santa Cruz Biotechnology. LPS (200 pg/ml), MTT and other reagents were purchased from Sigma Chemical. All reagents were tested for endotoxin using the high-sensitivity version of the Limulus Amebocyte Lysate assay (Biowhittaker) and were from the lots containing the lowest amounts of endotoxin available.

Spherical PMMA particles (Polysciences) 1–10 μm in diameter (6.0 μm mean diameter, 95% < 10 μm). The particles were sterilized in 70% ethanol and incubated overnight with shaking at 4 °C in sterile PBS with penicillin (100 units/ml) and streptomycin (100 μg/ml) at a concentration of 0.5 mg/ml (2×10^8^ particles/25 μl) until use. Adherent endotoxin on the particles (620 EU/ml) was measured using the high-sensitivity version of the Limulus Amebocyte Lysate Assay (Biowhittaker) in the presence of a β-glucan blocking reagent (Biowhittaker).

### Cell culture and cell viability assay

Raw264.7 cells, a murine macrophage cell line, were purchased from American Type Culture Collection. The cells were maintained in Dulbecco's modified Eagle's medium (DMEM) containing 50 g/ml streptomycin, 10% FBS (HyClone) and 50 units/ml penicillin at 37°C in a humidified atmosphere with 5% CO_2_. For all experiments, the cells were grown to 80–90% confluency and were subjected to no more than 20 cell passages. The Raw264.7 cells were incubated in 0.5 ml of RPMI-1640 supplemented with 10% FBS.

The cells were plated at a density of 5×10^4^ cells per well in 96-well plates to examine the effect of different concentration of UTI on activation of PMMA-stimulated Raw264.7 cells. Cells were serum-starved for 12 h and then UTI was added at 500, 5000 and 50000 units/ml to the culture medium 2 h before pretreatment of PMMA (0.5 mg/ml) with adherent LPS stimulation. Viable cells were stained with MTT (0.5 mg/ml) from CellTiter96® AQueous Non-Radioactive Cell Proliferation Assay (Promega) 12, 24 and 48 h after incubation of the cells. The culture (100 ul) from each well was transferred to a 96-well plate and the absorbance was read and recorded.

### Tartrate-resistant acid phosphatase staining

The presence of OC-like cells in LPS/PMMA-stimulated Raw264.7 cells was identified by tartrate-resistant acid phosphatase (TRAP) staining using a commercial kit (Sigma). LPS/PMMA-stimulated Raw264.7 cells were prepared and incubated in xylene for 30 s and washed with ethanol. Cells were incubated at 37°C for 1 h in 8 mM sodium tartrate, 2.2 mM Fast Garnet GBC and 100 mM acetate buffer (pH 5.2) containing 0.5 mM naphthol AS-BI phosphoric acid. The cells were then washed in several changes of distilled water, followed by counterstaining with a haematoxylin solution. The presence of dark purple staining granules in the cytoplasm was determined as the specific criterion for TRAP-positive cells. Positive TRAP localization was quantified by pixel area count 12, 24 and 48 h after incubated.

### Flow cytometric analysis

The Raw264.7 cells (1×10^5^ cells/ml) were cultured for 1 day in α-MEM supplemented with 10% FBS in the presence of soluble LPS (200 pg/ml) or PMMA (0.5 mg/ml) with adherent LPS, with or without UTI (500 or 5000 units/ml). Unstimulated OC precursors were identified as controls because Raw264.7 cells endogenously expressed receptor activation of nuclear factor kappa B (RANK). Cells were washed twice with PBS and incubated with an anti-RANK antibody conjugated with phycoerythrin. The labelled cells were analysed on a FACSCalibur (BD Bioscience). The ratio of RANK-positive cells among the total Raw264.7 cells was quantified using the labelled M1 line 48 h after incubated.

### Reverse transcriptase-polymerase chain reaction

Total RNA was extracted from cultured cells by using RNA-Bee (TEL-TEST). Complementary DNA was synthesized from the freshly isolated total RNA (1 mg) by using SuperScript™-II trans-criptase (Invitrogen) following the manufacturer's instructions. The synthesized cDNA was reacted and amplified with specific PCR primers in the presence of Taq polymerase (Hot Start Taq, Qiagen). The primer sequences are as follows: matrixmetallo-proteinases-9 (MMP-9) forward 5′-GGCGTGTCTGGAGATTCG-3′; MMP-9 reverse 5′-TA-CTGGAAGA TGTCGTGTGAG-3′; TNF-α forward 5′-GAG-TCCGGGCAGGTCTACTT T-3′; TNF-α reverse 5′-CAGGT-CACTGTGTCCCAGCATCT-3′; IL-6 forward 5′-CAGGTCA-CTGTGTCCCAGCATCT-3′; IL-6 reverse 5′-TCTGAC CAC-AGTGAGGAATGTCCAC-3′; RANK forward 5′-CTGCCTC-TGGGA ACGTGACT-3′; RANK reverse 5′-GCGAGGTCTG-GCTGACATAC-3′; cathepsin K forward 5′-CTGAAGATGC-TTTCCCATATGTGGG-3′; cathepsin K reverse 5′-GTCCCT-CACCCTCCCAAAAGG-3′; GAPDH forward 5′-CCAATGT-GTCCGTCGTGGAT-3′; GAPDH reverse 5′-TGC TGTTGA-AGTCGCAGGAC-3′. The amplified PCR products were separated by electrophoresis in 1.5% agarose gels. The PCR products in the gels were visualized by SYBR safe™ (Invitrogen) and scanned using AlphaImager®.

### Sample preparation and immunoblot analysis

Cells were lysed in 1 mM DTT, 1% Triton X-100, protease inhibitors (Roche), 200 mM NaCl and TNT buffer containing 20 mM Tris/HCl (pH 7.5). The protein content of the samples was measured using Pierce reagents, following the manufacturer's protocol. Protein samples of 20 μg were subjected to SDS/PAGE, and the proteins were then transferred to a PVDF membrane (100 V, 1 h, 4°C). The membranes were then incubated with anti-I-κB, anti-ERK (1/2), anti-MEK, anti-JNK, p-38 and anti-β-actin antibodies at optimal dilutions in 5% (v/v) skim milk solution supplemented with 0.01% (v/v) azide overnight at 4°C. The blots were washed in TTBS (50 mM NaCl, 10 mM Tris/HCl, 0.25% Tween 20) and incubated with an appropriate secondary antibody for 30 min at room temperature. The immunoreactive proteins were visualized using enhanced chemiluminescence reagents (GE Healthcare).

### Murine osteolysis model

In all, 40 male Sprague–Dawley rats (12 weeks old, 450±20 g) were used to establish a simple and reproducible animal model for particle-induced osteolysis. The rats were divided evenly into four groups at random: blank control group, PMMA-induced group, UTI (500 units/kg per day) group and UTI (5000 units/kg per day) (*n*=10 per group). Bilateral femurs of the hind leg of all experimental rats underwent the surgical procedure, which was performed under strictly aseptic conditions. Pentobarbital sodium solution was used for anaesthesia by intraperitoneal injection (Kyoritsu-Seiyaku, 50 mg/kg body weight). A lateral parapatellar approach was used to expose the knee joint, and a bone canal (10 mm long, parallel to the long axis of femur) was created with a 1.5 mm diameter drill in the intercondylar fossa. In the PMMA-induced group and UTI group, 100 μl PMMA particle suspension (0.5 mg/ml) with adherent LPS was injected into the canal. In the blank control group, an equal volume of saline solution was injected. The incision was closed using a 4–0 Ethibond suture. UTI group received 500 or 5000 units/kg UTI daily by intraperitoneal injection from day 1 to 6 weeks post-operatively. Animals were allowed to move without restriction in their cages after surgery. In the second weeks postoperatively, after anaesthesia with pentobarbital sodium solution, 100 μl vehicle saline solution was re-injected into the knee joint cavity of animals in the blank control group with a 1 ml syringe, and an equal volume of PMMA particle suspension with adherent LPS was used in the PMMA-induced and UTI groups. The procedures were approved by the Institutional Ethics Committee of the Medical School of Yangzhou University. All animals were killed with an overdose intraperitoneal injection of pentobarbital sodium 3 weeks after surgery. Bilateral femurs of the hind leg were collected for analyses.

### Histopathology

Tissue samples were fixed in 4% polyoxymethylene (pH 7.4) for 24 h, after decalcification in 10% EDTA. The specimens were processed for dehydration in graded alcohol, cleared in dimethyl benzene and embedded in paraffin. Tissue sections (6 μm) were stained with haematoxylin and eosin (HE) to evaluate the inflammatory condition of interfacial membrane and the erosion of the implanted calvarial bone slices. The stained sections were examined under a light microscope (Olympus DP70; Olympus Optical) and digital photo-micrographs were captured and analysed using a computerized image analysis system with Image-Pro Plus software, version 6.0 (Media Cybernetics). Interfacial membrane thickness (mm) and the total numbers of infiltrated cells (cells/mm^2^) around the implanted were measured using digital image analysis. Four separate sections/specimen were analysed in a blinded fashion. Interfacial membrane thickness was measured at six points on each section and six random 100 μm longitudinal areas were selected to count total cells based upon nucleus count.

### Micro-computed tomography

Bone microarchitecture in distal femur was scanned by eXplore Locus SP Pre-Clinical Specimen micro-computed tomography (micro-CT; GE Healthcare). The reconstruction and 3D quantitative analyses were performed by the desktop micro-CT system. The scanning regions were confined to the distal metaphysis, extending 2.0 mm proximally from the proximal tip of the primary spongiosa for the cancellous portion. The 3D indices analysed in the defined region of interest (ROI) were bone mineral density (BMD), trabecular number (Tb.N.), trabecular separation (Tb.Sp.), relative bone volume over total volume (BV/TV, %) and structure model index (SMI) of the cancellous bones. The operator conducting the scan analysis was blinded to the treatments associated with the specimens.

### Isolation of macrophages from the femurs of animal models

Macrophages were isolated from the femurs of murine osteolysis model as previously described [20]. Briefly, cells were flushed from femurs and incubated in differential medium consisting of RPMI-1640 media (Invitrogen), 30% macrophage-colony stimulating factor, 13% FBS, 5% horse serum and 0.5% glucose for 7 days to allow proliferation and differentiation. At confluence, cells were harvested with trypsine digestion. Cells were then seeded in culture dishes in HEPES-buffered phenol red-free RPMI-1640 media (Cellgro) supplemented with 10% charcoal dextran-stripped FBS (HyClone) and 1% penicillin/streptomycin and 1% sodium pyruvate (Invitrogen) to allow adherence.

### ELISA

The conditioned medium were collected, filtered through a 0.22 mm filter and then assayed for some inflammation-related cytokines. MMP-9, TNF-α, IL-6, RANK and cathepsin K were determined using ELISA kit (Pierce). All above tests were accomplished according to the manufacturer's instructions.

### Statistical analysis

Statistical analyses were conducted using SPSS for Windows (Release 14.0 K, SPSS). Multiple comparison tests among different dose groups were analysed by one-way ANOVA. The data were expressed as mean ± S.D. The criterion for statistical significance was set at *P*< 0.05 or *P*< 0.01.

## RESULTS

### Effect of UTI on cell viability in PMMA-stimulated Raw264.7 cells

**Figure 1:**
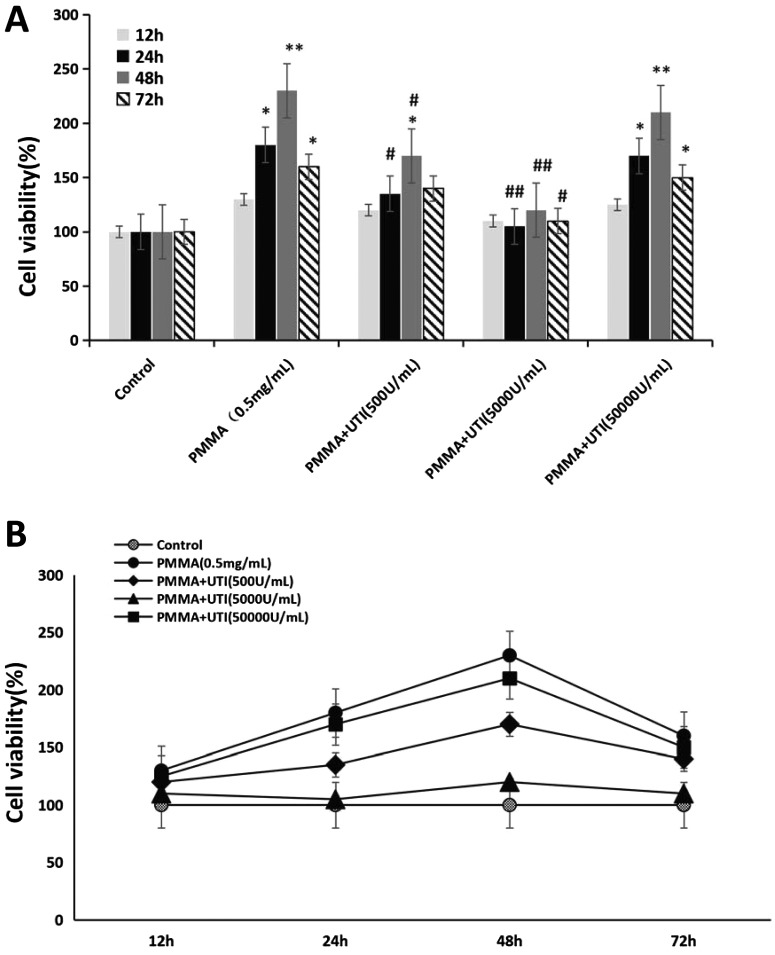
UTI inhibited the cell viability of PMMA-stimulated Raw264.7 cells Raw264.7 cells were treated with or without various concentration (500, 5000 or 50000 units/ml) of UTI for 3 h prior to being treated with 0.5 mg/ml PMMA with adherent LPS for 12, 24, 48 and 72 h. After incubation, cell viability was assessed by the MTT analysis among different groups (**A**), and among different time points (**B**) respectively. Data are presented as mean ±S.E.M. from three independent experiments. ^*^, *P*<0.05 and ^**^, *P*<0.01 compared with control; ^#^, *P*<0.05 and ^##^, *P*<0.01 compared with PMMA group.

Whether UTI has some effects on cell viability in PMMA-stimulated Raw264.7 cells is unknown. In the present study, we performed MTT assay to examine the effect of various concentration of UTI (500, 5000 and 50000 units/ml) on cell viability in PMMA-stimulated Raw264.7 cells. As shown in Figure 1(A), cell viability (%) of PMMA group was significantly increased in Raw264.7 cells compared with that of the control. Though reduction in cell viability by PMMA was significantly changed by pretreatment with UTI (500 or 5000 units/ml) in a dose-dependent manner, cell viability (%) did not show the significant difference after pretreatment with 50000 units/ml UTI compared with that of the PMMA group. Furthermore, the values of cell viability in Raw264.7 cells from each group reached the peak at 48 h after cultured, and then was on the decline (Figure 1B). These results suggested that the concentration of UTI (50000 units/ml) almost has no effects on cell viability in PMMA-stimulated Raw264.7 cells. Thus, the concentrations of UTI (500 and 5000 units/ml) were chosen for examining the anti-inflammatory effects of UTI in subsequent experiments.

### Effect of UTI on the expression of positive TRAP cells in LPS/PMMA-induced Raw264.7 cells

**Figure 2:**
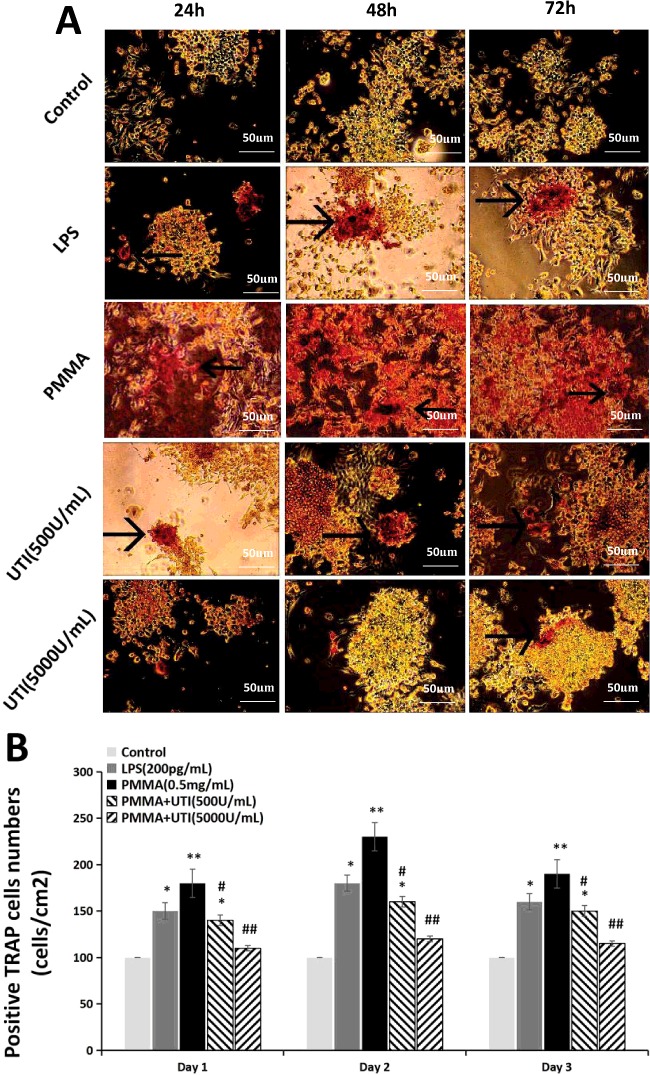
UTI suppressed the expression of TRAP in LPS/PMMA-induced Raw264.7 cells in a dose-dependent manner The cells were stimulated with alone LPS (200 pg/ml) or treated with or without various concentration (500 and 5000 units/ml) of UTI for 3 h prior to being treated with 0.5 mg/ml PMMA with adherent LPS for 24, 48 and 72 h. (**A**) Representative TRAP staining images of Raw264.7 cells from each group. The presence of dark purple staining granules in the cytoplasm was determined as the specific criterion for TRAP-positive cells (the black arrow points). (**B**) TRAP-positive cells number (cells/cm^2^) shown in (A) was quantified by pixel area count. Data are presented as mean±S.E.M. from three independent experiments. ^*^, *P*<0.05 and ^**^, *P*<0.01 compared with control; ^#^, *P*<0.05 and ^##^, *P*<0.01 compared with PMMA group.

To investigate the effect of UTI on OC activation and formation of mature OCs, we examined the expression levels of positive TRAP cells in LPS/PMMA-induced Raw264.7 cells 24, 48 and 72 h after cultured from each group using TRAP staining methods (Figure 2A). Positive TRAP levels of Raw264.7 cells in LPS group and PMMA group appeared higher compared with those in the control group. In contrast, treatment with UTI (500 units/ml) or UTI (5000 units/ml) have a significantly reversing trend to the increased positive TRAP levels in Raw264.7 cells in a dose-dependent manner, but not in a time-dependent manner (Figure 2B). Furthermore, the values of positive TRAP levels of Raw264.7 cells in PMMA group reached the peak at 48 h after cultured, and then was on the decline (Figure 2B). These results suggested that treatment with UTI (500 units/ml) or UTI (5000 units/ml) has the preventive effects in osteoclastogenesis of LPS/PMMA-stimulated Raw264.7 cells.

### Effect of UTI on proinflammatory mediators production in LPS/PMMA-stimulated Raw264.7 cells

**Figure 3:**
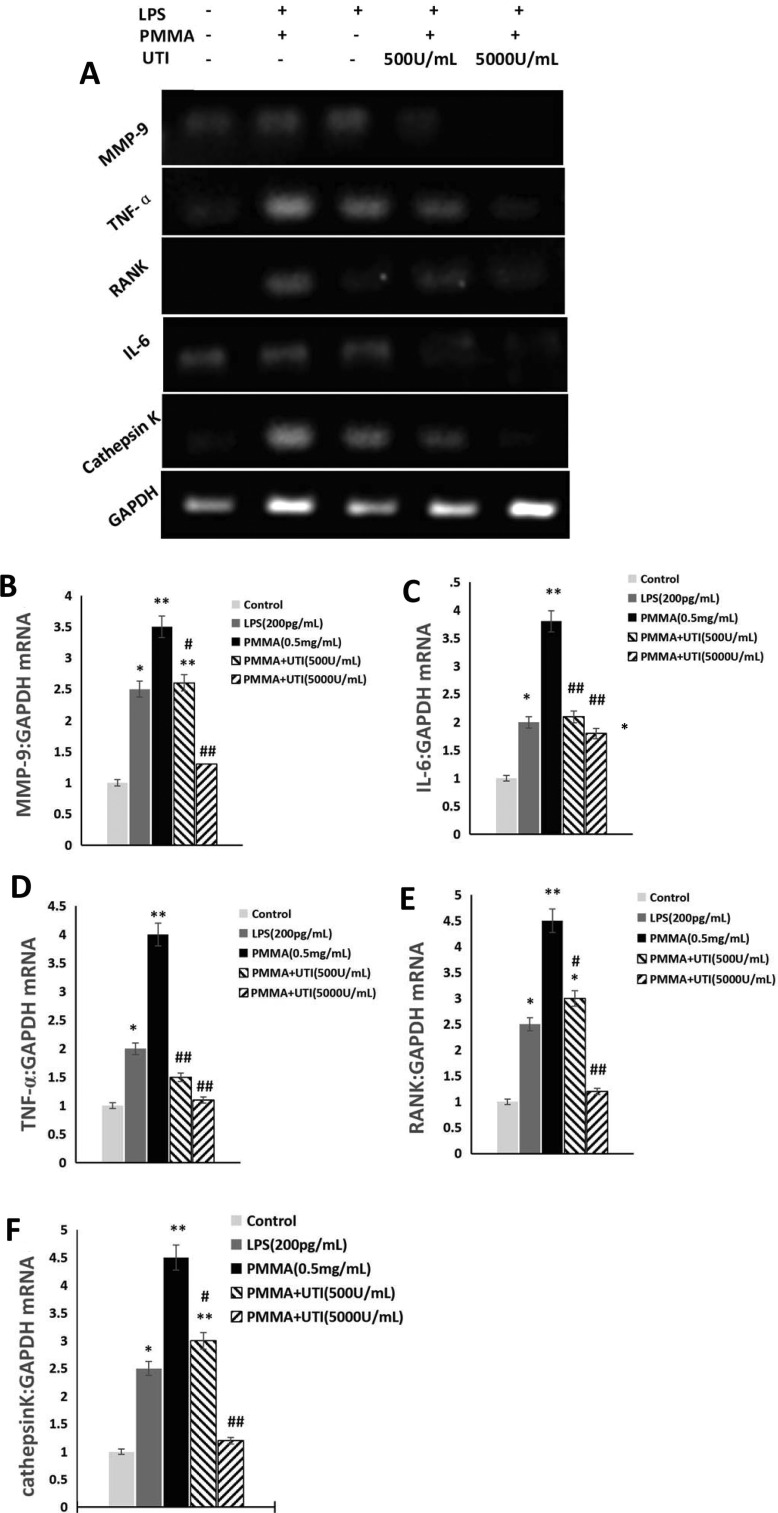
UTI decreased the expression levels of proinflammatory cytokines mRNA in LPS/PMMA-stimulated Raw264.7 cells in a dose-dependent manner The cells were stimulated with alone LPS (200 pg/ml) or treated with or without various concentration (500 and 5000 units/ml) of UTI for 3 h prior to being treated with 0.5 mg/ml PMMA with adherent LPS for 48 h. (**A**) Real time RT-PCR was performed to examine the expression levels of MMP-9, TNF-α, RANK, IL-6 and cathepsin K mRNA in Raw264.7 cells from each group. (**B**) Quantification of the relative ratio of MMP-9 (**B**), IL-6 (**C**), TNF-α (**D**), RANK (**E**) and cathepsin K (**F**) mRNA shown in (A). Data are presented as mean±S.E.M. from three independent experiments. ^*^, *P*<0.05 and ^**^, *P*<0.01 compared with control; ^#^, *P*<0.05 and ^##^, *P*<0.01 compared with PMMA group.

**Figure 4:**
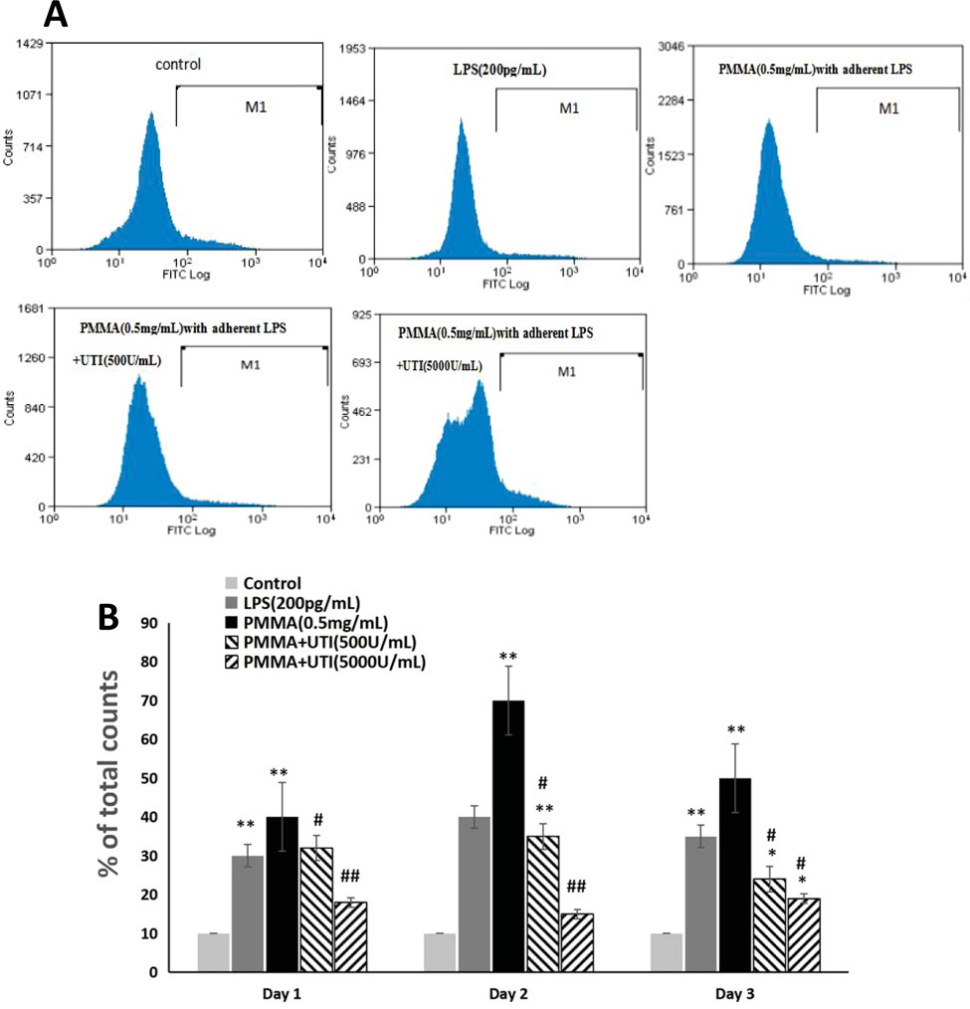
UTI inhibited the levels of RANK-positive cells in a dose-dependent manner in LPS/PMMA-induced Raw264.7 cells The cells were stimulated with alone LPS (200 pg/ml) or treated with or without various concentration (500 and 5000 units/ml) of UTI for 3 h prior to being treated with 0.5 mg/ml PMMA with adherent LPS for 24 h. (**A**) Flow cytometry was performed to examine the levels of RANK-positive cells in Raw264.7 cells from each group. (**B**) The ratio of RANK-positive cells among total nonadherent Raw264.7 cells was quantified by the labelled M1 line. Data are presented as mean ±S.E.M. from three independent experiments.^*^, *P*<0.05 and ^**^, *P*<0.01 compared with control; ^#^, *P*<0.05 and ^##^, *P*<0.01 compared with PMMA group.

MMP-9, TNF-α, RANK, IL-6 and cathepsin K mRNA levels were obtained to reflect the condition of proinflammatory cytokines production by real time reverse transcriptase-polymerase chain reaction (RT-PCR) (Figure 3A). Treatment with LPS (200 pg/ml) or PMMA (0.5 mg/ml) with adherent LPS exhibited significantly increased the expression levels of MMP-9 (Figure 3B), IL-6 (Figure 3C), TNF-α (Figure 3D), RANK (Figure 3E) and cathepsin K (Figure 3F) mRNA compared with the control group. More importantly, the effect of PMMA (0.5 mg/ml) with adherent LPS was more notable than that of LPS (200 pg/ml) on the expression levels of MMP-9, IL-6, TNF-α, RANK and cathepsin K (Figures 3B–3F) in LPS/PMMA Raw264.7 cells. In contrast, UTI (500 or 5000 units/ml) pretreatment markedly inhibited the expression levels of MMP-9, IL-6, TNF-α, RANK and cathepsin K (Figures 3B–3F) in a dose-dependent manner compared with PMMA-stimulated group. However, in the case of TNF-α and IL-6, no significant difference was shown between the 500 and 5000 units/ml UTI pretreatment group (Figures 3C and 3D). Furthermore, in our present study, the cell surface expression of the RANK protein (CD256) was analysed by FACS in Raw264.7 cells (Figure 4A). Similar with the results of RT-PCR, the effect of PMMA (0.5 mg/ml) with adherent LPS was more notable than that of LPS (200 pg/ml) on the cell surface expression of the RANK protein (CD256) in Raw264.7 cells. In contrast, UTI (500 or 5000 units/ml) pretreatment significantly suppressed the expression levels of RANK protein (CD256) in a dose-dependent manner compared with PMMA-stimulated group (Figure 4B). Furthermore, the values of expression levels of RANK protein (CD256) in Raw264.7 cells from PMMA group reached the peak at 48 h after cultured, and then was on the decline (Figure 4B). Collectively, these results indicate that UTI inhibited the secretion of proinflammatory mediators in LPS/PMMA-activated cells, which might result, at least in part, from the perturbation of common signalling pathways involving proinflammatory cytokines induction.

### Effect of UTI on NF-κB and MAPKs signalling pathway in Raw264.7 cells

**Figure 5:**
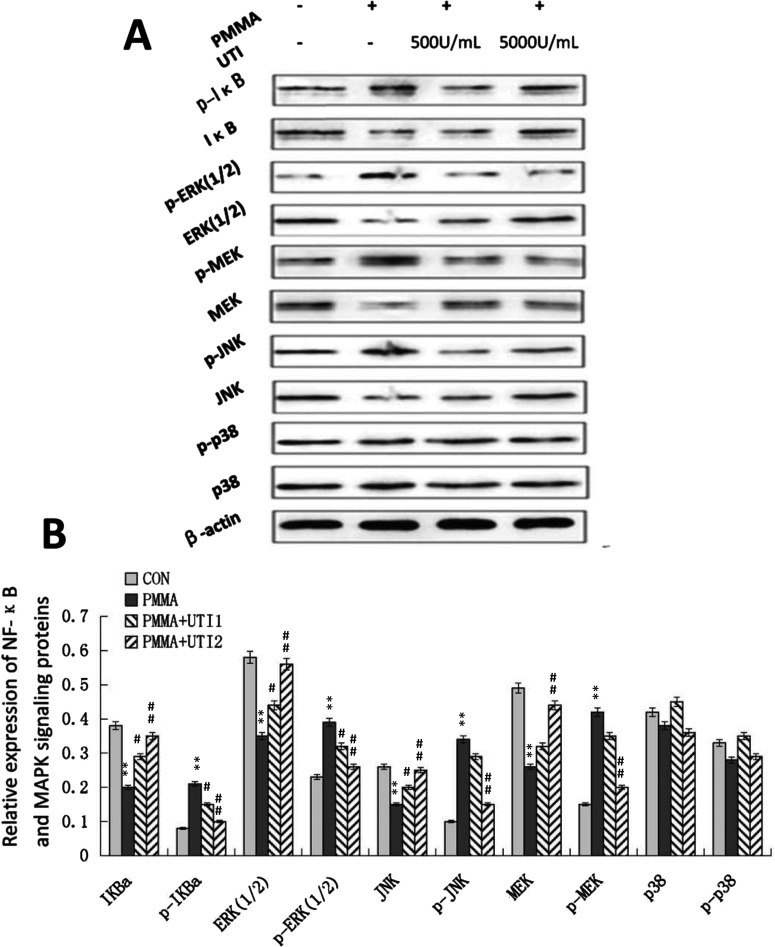
Activation of NF-κB and MAPK signalling pathway were suppressed by UTI in LPS/PMMA-induced Raw264.7 cells The cells were stimulated with treated with or without various concentration (500 and 5000 units/ml) of UTI for 3 h prior to being treated with 0.5 mg/ml PMMA with adherent LPS for 24 h. (**A**) Western blots was performed to examine the expression levels of NF-κB and MAPK signalling proteins in Raw264.7 cells from each group. (**B**) Quantification of the relative expression of NF-κB and MAPK signalling proteins. Data are presented as mean ±S.E.M. from three independent experiments. ^*^, *P*<0.05 and ^**^, *P*<0.01 compared with control; ^#^, *P*<0.05 and ^##^, *P*<0.01 compared with PMMA group.

Previous studies have suggested that UTI can inhibit LPS-induced TNF-α and subsequent IL-1β and IL-6 induction by macrophages, at least partly, through the suppression of MAPK signalling pathways such as ERK1/2, JNK and p38 *in vitro* or NF-κB in activation [19]. Thus, as a plausible molecular mechanism for UTI-mediated inhibition of inflammatory response, the effect of UTI on the NF-κB and MAPKs signalling pathway was explored via Western blot analysis (Figure 5A). The exposure of PMMA (0.5 mg/ml) with adherent LPS for 2 h facilitated the degradation of I-κBα, phosphorylation of I-κBα, nuclear accumulation of NF-κB, and significantly increased the expression of phosphorylated ERK (1/2), phosphorylated MEK and phosphorylated JNK. Interestingly, we observed significantly decreased phosphorylation of I-κB, ERK (1/2), MEK and JNK levels in Raw264.7 cells treated with UTI (500 or 5000 units/ml) in a dose-dependent manner (Figure 5B). Simultaneously, UTI (500 or 5000 units/ml) increased the expression levels of I-κBα, ERK (1/2), MEK and JNK in a dose-dependent manner. However, no obvious differences among four groups were observed on phosphorylation of p-38 and p-38 levels (Figure 5B). Collectively, these results indicated that the anti-inflammatory effect of UTI might be due to partial inhibition of the NF-κB and MAPKs signalling pathway.

### Inhibitory effect of UTI on PMMA-induced osteolysis *in vivo*

**Figure 6:**
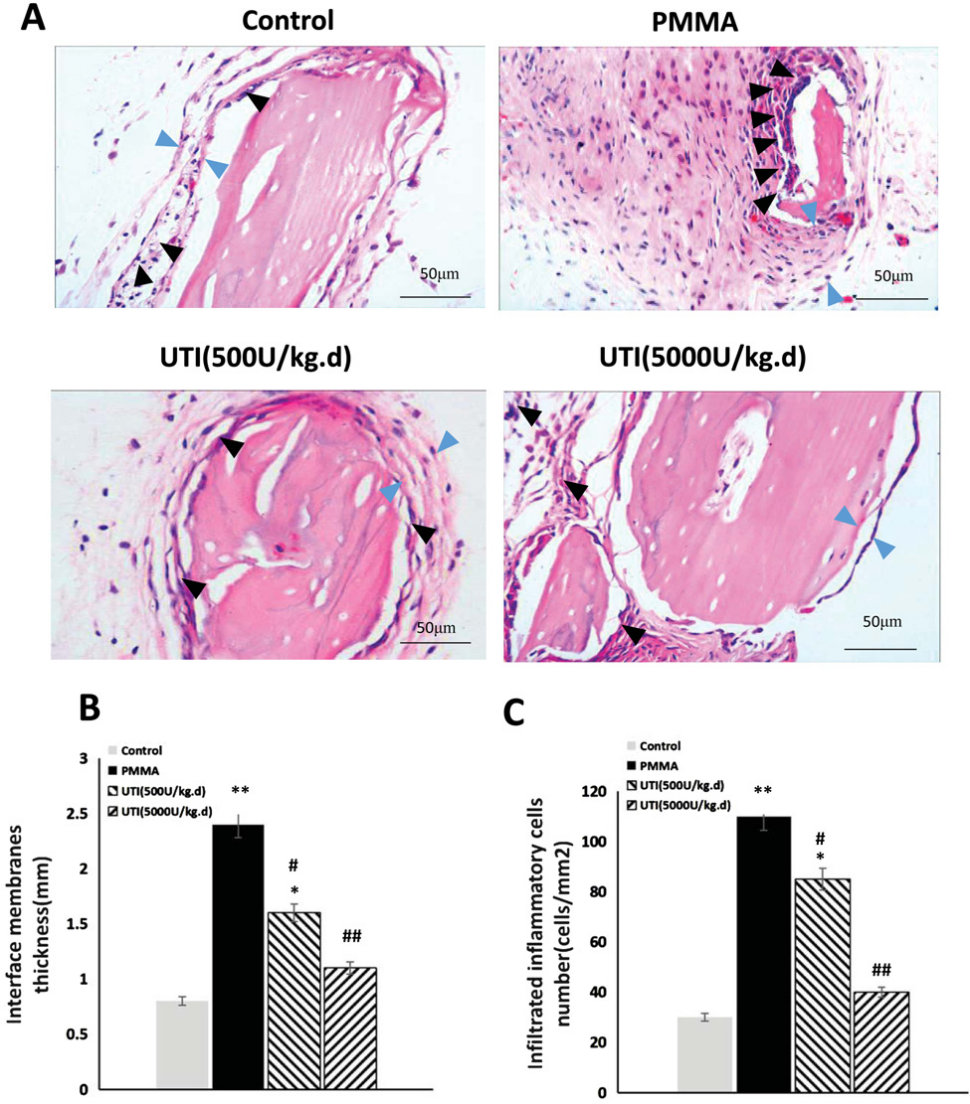
Total thickness of interfacial membrane (mm) and infiltrated inflammatory cells (cells/mm^2^) both were decreased by UTI in a dose-dependent manner in PMMA-induced murine osteolysis model The animal models were treated with or without various concentration of UTI (500 or 5000 units/kg i.p.) for 3 h prior to PMMA with adherent LPS implantation, and then UTI (500 or 5000 units/kg per day) was continuously injected (i.p.) once per day for 21 days. (**A**) Representative HE staining images of distal femur from each group. Total thickness of interfacial membrane (mm) and infiltrated inflammatory cells are indicated by blue and black arrowheads respectively. (**B**) Total thickness (mm) and (**C**) infiltrated inflammatory cells (cells/mm^2^) were quantified using automated image analyser. All values are expressed as mean ± S.E.M. *n*=10 mice per group. ^*^, *P*<0.05 and ^**^, *P*< 0.01 compared with control; ^#^, *P*<0.05 and ^##^, *P*<0.01 compared with PMMA group.

**Figure 7:**
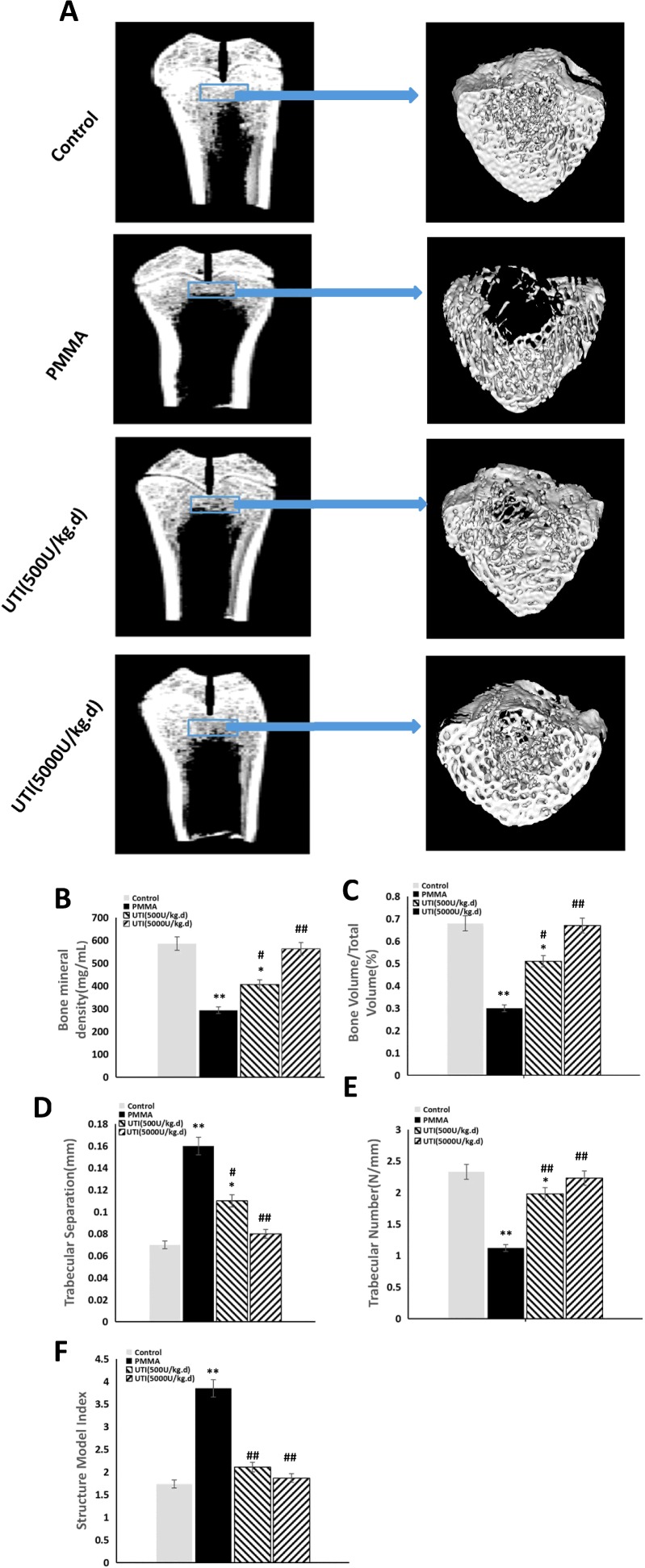
UTI inhibited the bone osteolysis in PMMA-induced murine osteolysis model The animal models were treated with or without various concentration of UTI (500 or 5000 units/kg i.p.) for 3 h prior to PMMA with adherent LPS implantation, followed by UTI (500 or 5000 units/kg) being injected (i.p.) once per day for 21 days. (**A**) Representative coronal section views of distal femur using micro-CT from each group (left) and the reconstructed images with 3D (right). The reconstructed sites are indicated by blue bar frames (left) and the bone osteolysis sites are indicated by white arrows (right). (**B**) BMD (mg/ml), (**C**) BV/TV (%), (**D**) Tb.Sp. (mm), (**E**) Tb.N. (N/mm) and (**F**) SMI of each sample were measured. All values are expressed as mean ± S.E.M. *n*=10 mice per group. ^*^, *P*<0.05 and ^**^, *P*<0.01 compared with control; ^#^, *P*<0.05 and ^##^, *P*<0.01 compared with PMMA group.

**Figure 8:**
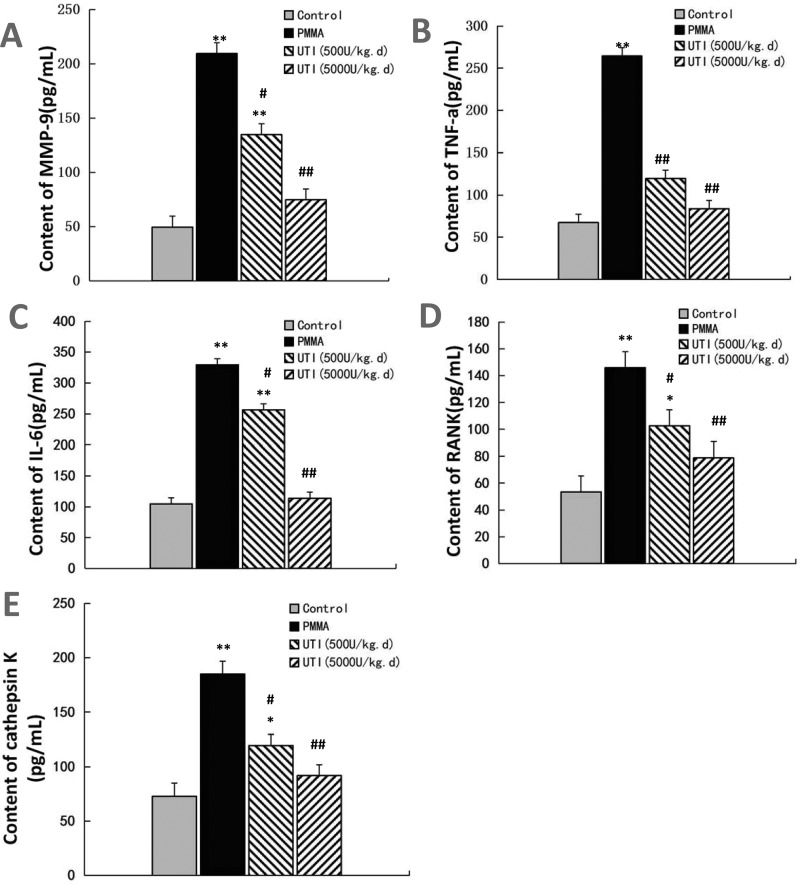
UTI suppressed the expression levels of proinflammatory cytokines in a dose-dependent manner in PMMA-induced murine osteolysis model Macrophages were isolated from the femurs of murine osteolysis model at the end of the experimental time line, and the conditioned medium from each group were collected, filtered through a 0.22 mm filter, and then assayed for some inflammation-related cytokines. MMP-9 (**A**), TNF-α (**B**), IL-6 (**C**), RANK (**D**) and cathepsin K (**E**) were quantified by ELISA. Data are presented as mean ± S.E.M. from three independent experiments. ^*^, *P*<0.05 and ^**^, *P*<0.01 compared with control; ^#^, *P*<0.05 and ^##^, *P*<0.01 compared with PMMA group.

To explore anti-inflammatory effect of UTI *in vivo*, the murine osteolysis model of distal femur was used, which is a commonly used model for studying AL. HE staining analysis of interfacial membrane around the implanted were carried out to validate the *in vivo* anti-inflammatory effect of UTI (Figure 6A). Marked increases of the thicknesses of interfacial membrane around the implanted were detected as results of PMMA-induced inflammatory changes, and notable increases of infiltrated inflammatory cells were also detected in interfacial membrane tissues as shown in Figure 6(A). In contrast, the thicknesses of interfacial membrane of UTI-treated groups (500 or 5000 units/kg per day i.p.) was significantly reduced by −16.32% and −28.89% compared with PMMA-treated group respectively (Figure 6B). Moreover, the numbers of infiltrated inflammatory cells in interfacial membrane tissues also were significantly decreased by −28.27% and −58.68% in UTI-treated groups (500 or 5000 units/kg per day i.p.) respectively (Figure 6B). Thus, UTI played a critical role in the significant reduction in these inflammatory changes in PMMA-induced murine osteolysis model. In addition, the osteolysis induced by the implantation of PMMA with adherent LPS was examined by micro-CT with 3D reconstruction. It was demonstrated that PMMA-induced osteolysis was suppressed by cotreatment with UTI (500 or 5000 units/kg per day i.p.) as shown in Figure 7(A). Quantitative analysis of bone parameters further confirmed that UTI significantly ameliorated the BMD (Figure 7B), BV/TV (bone volume/total volume) (Figure 7C) and Tb.N. (Figure 7E), and decreased the Tb.Sp. (Figure 7D) and SMI (Figure 7F) that induced by PMMA in a dose-dependent manner. In order to further confirm the anti-inflammatory effect of UTI *in vivo*, macrophages were isolated from the femurs of murine osteolysis model at the end of the experimental time line, and some inflammation-related cytokines from each group were assayed by ELISA. It was shown that the UTI-treated group (500 or 5000 units/kg per day) significantly suppressed the expression levels of MMP-9, IL-6, TNF-α, RANK and cathepsin K (Figures 8A–8E) in a dose-dependent manner compared with PMMA-induced group.

Collectively, these results indicate that UTI inhibited the bone osteolysis in PMMA-induced murine osteolysis model.

## DISCUSSION

The generation of wear particles is an inevitable result of normal usage of TJRs [1]. Although clinical success and increasing popularity in the surgical population have driven its indications to more and more young and physically demanding patients, wear particle-induced PPO and consequent AL, so-called ‘particle disease’, has been the most common cause of revision of major arthroplasties, which remains a major problem for these patients. The term ‘particle disease’ was coined by Dr William Harris to stress the importance of wear particles generated by a prosthesis for induction of host response [14,21]. The process of AL is believed to begin with the generation of wear particles from implant surfaces which are phagocytosed by immune cells leading to inflammatory cytokine release (such as IL-6, TNF-α, IL-1β and MMPs) and subsequent osteolysis [2,5,22–28]. However, it is also supported by many clinical studies that the bacterial PAMPs, of which the best-studied is LPS, may contribute to AL of orthopaedic implants [9,10]. PAMPs probably exist in periprosthetic tissue of some patients with AL whose sources can come from the bacterial biofilm that is often found on implants retrieved from patients despite the lack of any clinical signs of infection or the systemic circulation or the implants themselves [12,13]. Together with previous studies, LPS may play a vital role in PPO and AL. To date, strategies for the treatment of wear debris-induced osteolysis have been shown to be ineffective in humans and may cause adverse effects on other organs [15]. Such drugs as TNF-α inhibitor and bisphosphonates may lead to infection, pathologic femoral fractures, the impairment of fracture healing, mandibular lesions and other adverse events [16]. Thus, new strategies and biologics for wear particle-induced osteolysis are urgently needed.

UTI is a multivalent Kunitz-type serine protease inhibitor found in human urine and blood [17,18]. Various serine proteases such as trypsin, chymotrypsin, neutrophil elastase and plasmin are reportedly inhibited by UTI whose broad-spectrum inhibitory activity does not overlap completely [19]. Based on the multivalent nature of protease inhibition, UTI appears to prevent organ injury by inhibiting the activity of these proteases [17–19].

Beyond its inhibition of inflammatory proteases mentioned above, UTI exhibits anti-inflammatory activity and suppresses the infiltration of neutrophils and release of elastase and chemical mediators from them [29,30]. As well, UTI reportedly inhibits the production of TNF-α and IL-1 in LPS-stimulated human monocytes and LPS- or neutrophil elastase-stimulated IL-8 gene expression in HL60 cells or bronchial epithelial cells *in vitro* [31]. Furthermore, it was reported that UTI effectively inhibited the increased expression of MMP-2, MMP-3, NOS in degenerated NP cells induced by IL-1β *in vitro* which suggests that UTI may potentially be useful for clinical therapy of intervertebral disc degeneration [32]. Together with all previous studies, it has been shown that UTI has the therapeutic potential to treat LPS-related inflammatory diseases and subsequent organ damage with less side effects. However, no report has been made on its use for LPS-related PPO and subsequent AL.

MMP-9 may play a key role in facilitating orthoclastic bone resorption by degradation of extracellular matrix macromolecules present around and on the surface of the bone trabeculae [33]. In the present study, UTI pretreatment significantly blocked the secretion of MMP-9, RANK and cathepsin K in a dose-dependent manner compared with LPS/PMMA-stimulated cells, though various concentration of UTI (500 or 5000 units/ml) pretreatment showed a similar reduction in the case of TNF-α and IL-6. These results indicate that UTI inhibited the secretion of proinflammatory mediators in LPS/PMMA-activated cells. In addition, the murine osteolysis model of distal femur is believed to mimic the pathological processes of wear particle-induced osteolysis in humans [34,35]. The high dose of UTI (5000 units/kg per day) was chosen to be used in animal models as physiologically relevant. We have found that PMMA-induced acute inflammatory changes and inflammatory cell infiltrations were significantly suppressed by treatment with various concentration of UTI in a dose-dependent manner in a murine model of femur resorption. Moreover, all experimental animals in the current study had not shown any symptoms at the end of the experimental time line. Collectively, these results have shown that UTI may play an inhibitory role in the inflammatory pathological response in osteoclastogenesis and oeteolysis, thus enhancing the osteogenesis and delaying the development of particle disease.

Recent studies have shown that PMMA particles accelerate osteoclastogenesis of pre-OCs through amplification of various signals including NF-κB, MAP kinases and nuclear factor of activated-T-cells (NFAT) signal transduction pathways [36]. It was also demonstrated that UTI inhibits LPS-induced TNF-α and subsequent IL-1β and IL-6 induction by macrophages, at least partly, through the suppression of MAPK signalling pathways such as ERK1/2, JNK and p38 *in vitro* [37,38]. Whether the inflammatory role of UTI is related with NF-κB and MAPKs signalling pathways is unknown. In the present study, we tested the effect of UTI on the NF-κB and MAPKs signalling pathways in LPS/PMMA-treated Raw264.7 cells, and found that both NF-κB and MAPKs mediated the activation of LPS/PMMA-treated Raw264.7 cells. Importantly, though UTI had little effect on phosphorylation of p-38, various concentration of UTI (500 or 5000 units/ml) decreased the phosphorylation of I-κB, ERK (1/2), MEK and JNK in a dose-dependent manner in LPS/PMMA-stimulated Raw264.7 cells. Thus, the anti-inflammatory effect of UTI might be due to partial inhibition of the NF-κB and MAPKs signalling pathway.

## CONCLUSIONS

Taken together, although UTI inhibits the osteoclastogenesis and oeteolysis *in vitro* and *in vivo*, its pharmacological research and ultimate human testings are further needed. In the present study, UTI significantly inhibited the expression of inflammatory mediators such as MMP-9, RANK and cathepsin K in a dose-dependent manner in LPS/PMMA-stimulated Raw264.7 cells. As a plausible molecular mechanism, increased degradation and phosphorylation of I-κB, ERK (1/2), MEK and JNK by RANKL/PMMA were partly blocked by UTI treatment. More importantly, UTI treatment attenuated the inflammatory osteolysis reaction in PMMA-induced murine osteolysis models. These results demonstrate that UTI has an anti-inflammatory therapeutic potential in the prevention of PPO and consequent AL, which may result from the inhibition of the expression of proinflammatory mediators via NF-κB and MAPKs partial inactivation. UTI could be further developed for prevention and treatment of particle disease as a potential drug with less adverse events. Further studies on UTI are needed to throw light on how it may be used clinically for prevention and treatment of particle disease.

## Abbreviations

AL: aseptic loosening
BMD: bone mineral density
BV/TV: bone volume/total volume
HE: haematoxylin–eosin
IL-1α: interleukin-1α
IL-1β: interleukin-1β
IL-6: interleukin-6
LPS: lipopolysaccharide
MAPK: mitogen activated protein kinase
micro-CT: micro-computed tomography
MMP-9: matrixmetallo-proteinases-9
NF-κB: nuclear factor kappa B
OC: osteoclast
PAMP: pathogen-associated molecular pattern
PMMA: polymethyl-methacrylate
PPO: peri-prosthetic osteolysis
RANK: receptor activation of nuclear factor kappa B
RT-PCR: reverse transcriptase-polymerase chain reaction
SMI: structure model index
Tb.N.: trabecular number
Tb.Sp.: trabecular separation
Tb.Th.: trabecular thickness
TJR: total joint replacement
TNF-α: tumour necrosis factor-α
TRAP: tartrate-resistant acid phosphatase
UTI: urinary trypsin inhibitor

## References

[1] Deirmengian G.K., Zmistowski B., O'Neil J.T., Hozack W.J. (2002). Management of acetabular bone loss in revision total hip arthroplasty. J. Bone Joint Surg. Am..

[2] Greenfield E.M., Bi Y., Ragab A.A., Goldberg V.M., Van De Motter R.R. (2002). The role of osteoclast differentiation in aseptic loosening. J. Orthop. Res..

[3] Zhang Y., Hou C., Yu S., Xiao J., Zhang Z., Zhai Q., Chen J., Li Z., Zhang X., Lehto M. (2012). IRAK-M in macrophages around septically and aseptically loosened hip implants. J. Biomed. Mater. Res. A.

[4] Gallo J., Vaculova J., Goodman S.B., Konttinen Y.T., Thyssen J.P. (2014). Contributions of human tissue analysis to understanding the mechanisms of loosening and osteolysis in total hip replacement. Acta Biomater..

[5] Purdue P.E., Koulouvaris P., Potter H.G., Nestor B.J., Sculco T.P. (2007). The cellular and molecular biology of periprosthetic osteolysis. Clin. Orthop. Relat. Res..

[6] Nakashima T., Hayashi M., Fukunaga T., Kurata K., Oh-Hora M., Feng J.Q., Bonewald L.F., Kodama T., Wutz A., Wagner E.F. (2011). Evidence for osteocyte regulation of bone homeostasis through RANKL expression. Nat. Med..

[7] Anderson J.M., Rodriguez A., Chang D.T. (2008). Foreign body reaction to biomaterials. Semin. Immunol..

[8] Boyle W.J., Simonet W.S., Lacey D.L. (2003). Osteoclast differentiation and activation. Nature.

[9] Greenfield E.M., Bi Y., Ragab A.A., Goldberg V.M., Nalepka J.L., Seabold J.M. (2005). Does endotoxin contribute to aseptic loosening of orthopaedic implants?. J. Biomed. Mater. Res. B Appl. Biomater..

[10] Greenfield E.M., Bechtold J. (2008). What other biologic and mechanical factors might contribute to osteolysis?. J. Am. Acad. Orthop. Surg..

[11] Nakashima T., Takayanagi H. (2011). New regulation mechanisms of osteoclast differentiation. Ann. N.Y. Acad. Sci..

[12] Wang J., Mazza G. (2002). Inhibitory effects of anthocyanins and other phenolic compounds on nitric oxide production LPS/IFN-gamma-activated RAW 264.7 macrophages. J. Agric. Food Chem..

[13] Bi Y., Seabold J.M., Kaar S.G., Ragab A.A., Goldberg V.M., Anderson J.M., Greenfield E.M. (2001). Adherent endotoxin on orthopedic wear particles stimulates cytokine production and osteoclast differentiation. J. Bone Miner. Res..

[14] Gallo J., Vaculova J., Goodman S.B., Konttinen Y.T., Thyssen J.P. (2014). Contributions of human tissue analysis to understanding the mechanisms of loosening and osteolysis in total hip replacement. Acta Biomater..

[15] Lin T.H., Tamaki Y., Pajarinen J., Waters H.A., Woo D.K., Yao Z., Goodman S.B. (2014). Chronic inflammation in biomaterial-induced periprosthetic osteolysis: NF-kappaB as a therapeutic target. Acta Biomater..

[16] Goodman S.B., Gibon E., Pajarinen J., Lin T.H., Keeney M., Ren P.G., Nich C., Yao Z., Egashira K., Yang F., Konttinen Y.T. (2014). Novel biological strategies for treatment of wear particle-induced periprosthetic osteolysis of orthopaedic implants for joint replacement. J. R. Soc. Interface.

[17] Inoue K., Takano H., Yanagisawa R., Yoshikawa T. (2008). Protective effects of urinary trypsin inhibitor on systemic inflammatory response induced by lipopolysaccharide. J. Clin. Biochem. Nutr..

[18] Inoue K., Takano H. (2010). Urinary trypsin inhibitor as a therapeutic option for endotoxin related inflammatory disorders. Expert Opin. Investig. Drugs.

[19] Wu Y.J., Ling Q., Zhou X.H., Wang Y., Xie H.Y., Yu J.R., Zheng S.S. (2009). Urinary trypsin inhibitor attenuates heptic ischemia-reperfusion injury by reducing nuclear factor-kappa B activation. Hepatobiliary Pancreat. Dis. Int..

[20] Schilling D., Thomas K., Nixdorff K., Vogel S.N., Fenton M.J. (2002). Toll-like receptor 4 and Toll-IL-1 receptor domain-containing adapter protein (TIRAP)/myeloid differentiation protein 88 adapter-like (Mal) contribute to maximal IL-6 expression in macrophages. J. Immunol..

[21] Gallo J., Goodman S.B., Konttinen Y.T., Raska M. (2013). Particle disease: biologic mechanisms of periprosthetic osteolysis in total hip arthroplasty. Innate Immun..

[22] Kurtz S.M., Gawel H.A., Patel J.D. (2011). History and systematic review of wear and osteolysis outcomes for first-generation highly crosslinked polyethylene. Clin. Orthop. Relat. Res..

[23] Jablonski H., Kauther M.D., Bachmann H.S., Jager M., Wedemeyer C. (2015). Calcitonin gene-related peptide modulates the production of pro-inflammatory cytokines associated with periprosthetic osteolysis by THP-1 macrophage-like cells. Neuroimmunomodulation.

[24] Pearl J.I., Ma T., Irani A.R., Huang Z., Robinson W.H., Smith R.L., Goodman S.B. (2011). Role of the Toll-like receptor pathway in the recognition of orthopedic implant wear-debris particles. Biomaterials.

[25] O'Neill S.C., Queally J.M., Devitt B.M., Doran P.P., O'Byrne J.M. (2013). The role of osteoblasts in peri-prosthetic osteolysis. Bone Joint J..

[26] Fujii J., Niida S., Yasunaga Y., Yamasaki A., Ochi M. (2011). Wear debris stimulates bone-resorbing factor expression in the fibroblasts and osteoblasts. Hip Int..

[27] Wang R., Wang Z., Ma Y., Liu G., Shi H., Chen J., Dong L., Zhao J., Zhang J. (2013). Particle-induced osteolysis mediated by endoplasmic reticulum stress in prosthesis loosening. Biomaterials.

[28] Del Buono A., Denaro V., Maffulli N. (2012). Genetic susceptibility to aseptic loosening following total hip arthroplasty: a systematic review. Br. Med. Bull..

[29] Guo W., Li Z., Xie X., Qin T., Wu Y., Li Z., Chai J., Yi F., Tan T., Zhu H., Wang S. (2015). Urinary trypsin inhibitor attenuates acute lung injury by improving endothelial progenitor cells functions. Cell Physiol. Biochem..

[30] Zhang S., Yu R., Zhang Y., Chen K. (2014). Cytoprotective effects of urinary trypsin inhibitor on astrocytes injured by sustained compression. Mol. Biol. Rep..

[31] Deng Y., Kong J. (2015). Urinary trypsin inhibitor reduced inflammation response induced by hyperlipidemia. J. Cardiovasc. Pharmacol. Ther..

[32] Hua G., Haiping Z., Baorong H., Dingjun H. (2012). Effect of ulinastatin on the expression of iNOS, MMP-2, and MMP-3 in degenerated nucleus pulposus cells of rabbits. Connect. Tissue Res..

[33] Franco G.C., Kajiya M., Nakanishi T., Ohta K., Rosalen P.L., Groppo F.C., Ernst C.W., Boyesen J.L., Bartlett J.D., Stashenko P. (2011). Inhibition of matrix metalloproteinase-9 activity by doxycycline ameliorates RANK ligand-induced osteoclast differentiation *in vitro* and *in vivo*. Exp. Cell Res..

[34] Chen D., Li Y., Guo F., Lu Z., Hei C., Li P., Jin Q. (2015). Protective effect of p38 MAPK inhibitor on wear debris-induced inflammatory osteolysis through downregulating RANK/RANKL in a mouse model. Genet. Mol. Res..

[35] Cordova L.A., Trichet V., Escriou V., Rosset P., Amiaud J., Battaglia S., Charrier C., Berreur M., Brion R., Gouin F. (2015). Inhibition of osteolysis and increase of bone formation after local administration of siRNA-targeting RANK in a polyethylene particle-induced osteolysis model. Acta Biomater..

[36] Qu C.B., Bonar S.L., Hickman-Brecks C.L. (2015). NLRP3 mediates osteolysis through inflammation-dependent and -indepen-dent mechanisms. FASEB J..

[37] Neuerburg C., Wedemeyer C., Goedel J., Schlepper R., Hilken G., Schwindenhammer B., Schilling A.F., Jager M., Kauther M.D. (2015). The role of calcitonin receptor signalling in polyethylene particle-induced osteolysis. Acta Biomater..

[38] Mrazek F., Gallo J., Stahelova A., Petrek M. (2010). Functional variants of the P2RX7 gene, aseptic osteolysis, and revision of the total hip arthroplasty: a preliminary study. Hum. Immunol..

